# Evaluating general parent‐adolescent relations as a context for daily relationship processes and adolescent mood

**DOI:** 10.1111/jora.70224

**Published:** 2026-07-10

**Authors:** Lan Chen, Carlie J. Sloan, Damon E. Jones, Gregory M. Fosco

**Affiliations:** ^1^ Department of Human Development and Family Studies The Pennsylvania State University University Park Pennsylvania USA; ^2^ Research and Education Advancing Children's Health Institute Arizona State University Tempe Arizona USA; ^3^ Edna Bennett Pierce Prevention Research Center The Pennsylvania State University University Park Pennsylvania USA

**Keywords:** adolescent mood, daily diary, parent‐adolescent conflict, parent‐adolescent connectedness

## Abstract

General parent‐adolescent conflict and connectedness are well‐established predictors of adolescent well‐being. However, the processes by which historical relations contextualize daily fluctuations in parent‐adolescent conflict and connectedness provide insights into risk and protective dynamics within parent‐adolescent relationships across different timescales. Using baseline surveys and 21‐day daily diary data from a sample of 150 adolescents (*M*_age_ = 14.60, *SD*_age_ = 0.83, 61.3% female) and their parents (*M*_age_ = 43.4, *SD*_age_ = 6.9, 95% female) in two‐caregiver families, we examined whether general relationships moderate the daily associations between parent‐adolescent interactions and adolescent mood. Multilevel models revealed that the daily association between conflict and adolescent negative mood was stronger at higher levels of general conflict and weaker at higher levels of general connectedness. Importantly, the within‐person link between daily connectedness and adolescent mood remains robust, regardless of general relationship qualities. In sum, this study provides compelling evidence that simultaneously considering general qualities and daily parent‐adolescent relationships offers valuable implications for understanding adolescent mood.

The adolescent developmental period is characterized by changes across physical, cognitive, social, and emotional domains; however, relationships with parents remain the most salient (Núñez‐Regueiro & Núñez‐Regueiro, 2021). Parent‐adolescent relationships are a context for undermining or supporting adolescents' coping with changes in daily life. Parent‐adolescent conflict is a well‐documented risk factor for adolescent depressive symptoms and externalizing problems (Sallinen et al., 2007; Weymouth et al., 2016). Likewise, parent‐adolescent connectedness, characterized by feelings of support, love, and closeness, reduces the risk of adolescent internalizing, externalizing, and substance use outcomes (Boutelle et al., 2009; Fosco et al., 2012; Fosco, Brinberg, & Ram, 2021). Family‐based interventions have also provided experimental evidence that parent‐adolescent conflict and connectedness are drivers of adolescent well‐being (LoBraico et al., 2019; Van Ryzin et al., 2016).

Historically, past work has conceptualized parent‐adolescent conflict and connectedness primarily in terms of their general levels. *General levels* are considered typical perceptions of the overall parent‐adolescent relationship, which are relatively stable across contexts and time (Hamaker et al., 2007). Using global assessments, previous research has captured between‐person differences and central tendencies to understand which adolescents might experience more conflict or weaker connectedness with their parents and how these patterns relate to long‐term risks (e.g., Boutelle et al., 2009; Sallinen et al., 2007).

Another perspective focuses on how parent‐adolescent conflict and connectedness exhibit meaningful variation on a day‐to‐day basis (Fosco, Brinberg, & Ram, 2021; LoBraico et al., 2020). Changes that occur on a daily timescale are referred to as *daily variability*, to reflect the parent‐adolescent relationship on a specific day relative to their general relationships. Even in relationships characterized by high general connectedness, adolescents and parents experience “good days” and “bad days”, reflecting how connectedness has ups and downs even within generally close relationships (Fosco et al., 2019; Fosco, Brinberg, & Ram, 2021). Similarly, in relationships that are typically conflictual, the degree of daily conflict may vary, with adolescents experiencing days of either higher or lower conflict. Daily diary studies are particularly effective at capturing this daily variability, as they involve daily survey data collection (Bolger et al., 2003; Laurenceau & Bolger, 2005). Past research has documented that on days when relationships are more connected and less conflictual, adolescents experience greater positive moods, stronger feelings of being loved, and reduced negative emotions such as anger (Coffey et al., 2022; Fosco, Brinberg, & Ram, 2021; LoBraico et al., 2020; Vannucci et al., 2019). Considering daily parent‐adolescent conflict and connectedness provides an additional perspective for understanding adolescent well‐being.

## General conflict as a context of daily parent‐adolescent relationships

General levels of parent‐adolescent conflict may be a context that shapes the degree to which daily conflicts impact adolescents' mood and well‐being. This perspective is captured in a sensitization hypothesis. Research and theory about sensitization suggest that, rather than habituating to conflict, adolescents who have been exposed to a history of generally higher levels of family conflicts may become more sensitive to subsequent conflicts, evidenced by increases in reactivity to future conflicts (Cummings & Davies, 1994; Goeke‐Morey et al., 2013). Empirical studies support the sensitization hypothesis: children and adolescents exposed to intense interparental conflict show increased adverse outcomes to subsequent conflicts, resulting in heightened negative emotions, more adverse appraisals of conflict, increased maladaptive behaviors, and elevated cortisol levels (Cummings & Davies, 2002; David & Murphy, 2004; Davies et al., 1999; Goeke‐Morey et al., 2013; Grych, 1998; Koss et al., 2012). Longitudinal studies further validate this pattern, showing that adolescents exposed to high levels of interparental conflict early in development exhibited stronger negative reactions to conflict later in life (Davies et al., 2006, 2021). Recent work has extended this to the daily, within‐person level, finding that adolescents with greater exposure to general interparental conflict show elevated threat responses to daily conflict experiences (Sloan et al., 2022).

Building on the idea of sensitization to interparental conflict, similar dynamics may apply to parent‐adolescent conflict, as parent‐adolescent conflict is a salient feature of adolescent development (Branje, 2018) and may evoke a similar response in adolescence. Support for this notion is found in a recent study showing that, on days when adolescents experience parent‐adolescent conflict, the presence of general conflict could exacerbate the emotional reaction to daily conflict (Chiang et al. 2023). It is valuable to note that this study was conducted in Taiwan, where filial piety – values around obedience to one's parents – is a widely held cultural value, and as a result, less parent‐adolescent conflict is observed relative to Western cultures (Li et al., 2014). In contrast, adolescents in Western cultures often place greater value on autonomy and open expression of emotions, which may lead them to perceive parent‐adolescent conflicts as more threatening and more strongly linked to mood fluctuations. It is worth noting that in Chiang et al. (2023) study, which does report evidence of parent‐adolescent conflict sensitization, they did not replicate findings in studies with Western samples regarding interparental conflict sensitization. This divergence highlights how cultural context may shape conflict sensitization processes and underscores the need to examine parent‐adolescent conflict sensitization in Western samples. From a sensitization perspective, adolescents with a history of frequent, intense conflicts with their parents are expected to have stronger emotional responses to daily conflict events.

The current study further extends the sensitization hypothesis to evaluate whether general conflict may also shape the degree to which adolescents are reactive to daily variation in *positive* relationship quality ‐ parent‐adolescent connectedness. Although we are not aware of prior studies evaluating this relation, it is plausible that adolescents who experience higher levels of parent‐adolescent conflict may become especially attuned to daily departures from usual levels of connectedness with their caregivers (e.g., days when they feel less connected to caregivers than usual). Adolescents in generally conflictual relationships with their parents may become sensitized to changes in emotional cues like support and affection from their parents as a means of self‐protection (Fosco & LoBraico, 2019). Guided by a sensitization perspective, we expect that in relationships with high general parent‐adolescent conflict, adolescents may experience increased positive mood or reduced negative mood on days of high connectedness; conversely, even minor disruptions in daily connectedness may shape heightened negative mood or decreased positive mood (Mak et al., 2023).

## General connectedness as a context of daily parent‐adolescent relationships

A second general contextual factor for daily parent‐adolescent relationship processes may be parent‐adolescent connectedness. From an attachment perspective, adolescents who benefit from generally secure, trusting, and close relationships with their parents may be less affected by the day‐to‐day experiences, drawing instead on their established working models of the relationship (Bowlby, 1988; Moretti & Peled, 2004). From this view, adolescents who feel a general sense of closeness and connection with their parents may be less distressed by daily experiences of parent‐adolescent conflict and exhibit a lower degree of emotional reactivity to daily experiences of parent‐adolescent conflict (McKenna et al., 2022). One example from the interparental conflict literature suggests that in the context of high parent‐adolescent connectedness, daily experiences of interparental conflict are less disruptive for adolescents (Fosco, McCauley, & Sloan, 2021). Other work documents the buffering effect of positive parent‐adolescent relationships in daily interactions. For example, adolescents who experience higher levels of general parent‐adolescent communication or warmth tend to be less affected by daily conflicts and stressors compared to their peers (Lippold et al., 2016; Vannucci et al., 2019).

Likewise, following an attachment perspective, we expected that day‐to‐day changes in adolescents' connectedness with caregivers may be less distressing in the context of a generally high level of relationship connectedness to their parents. Adolescents who generally feel connected with their parents are likely to exhibit more stable emotional responses to daily fluctuations in connectedness. In contrast, adolescents who generally feel less connected to their parents may find daily variation more emotionally evocative – on days when they feel more connected to caregivers than usual, they may experience pronounced increases in positive mood and decreases in negative mood. Likewise, on days of less connectedness to caregivers, those with less secure general relationships may be particularly distressed. A parallel process was observed in a daily diary study focused on family cohesion (Fosco & Lydon‐Staley, 2020) – a generally high level of family cohesion moderated the relation between day‐to‐day variations in family cohesion and adolescents' daily mood. Specifically, adolescents with more generally cohesive families exhibited less emotional reactivity to daily changes in cohesion, with respect to depressed mood and positive well‐being. Together, extant research suggests that the daily association between parent‐adolescent relationships and adolescent mood may be weaker when adolescents experience higher general parent‐adolescent connectedness.

## The current study

**FIGURE 1:**
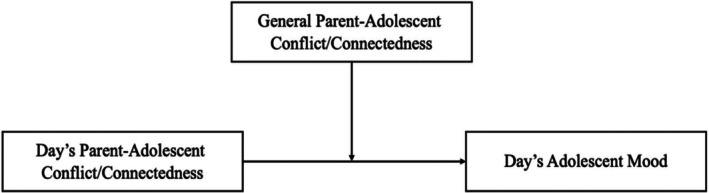
Conceptual Model.

Sensitization to conflict work focuses on understanding individual differences in their distress or emotional responses to conflict experiences (Cummings & Davies, 1994; Goeke‐Morey et al., 2013). Past work has documented that parent‐adolescent relationships are associated with adolescent mood at the daily level (Chiang et al., 2023; Fosco, Brinberg, & Ram, 2021). Additionally, daily diary studies using lagged analyses – including those explicitly testing bidirectional effects– have found that prior‐day parent‐adolescent conflict is associated with next‐day adolescent mood (Bülow et al., 2022; Fosco et al., 2025; LoBraico et al., 2020). Consistent with prior research testing the sensitization hypothesis (Chiang et al., 2023; Sloan et al., 2022), the current study used baseline and 21‐day daily diary data to examine how adolescents' general relationship perceptions shape daily linkages between relational experiences and mood (See Figure 1). We focused specifically on adolescent‐reported baseline measures because the sensitization framework emphasizes that adolescents' subjective perceptions and appraisals constitute the sensitizing context that shapes their reactivity to daily events (Grych & Fincham, 1990; Sloan et al., 2022). Baseline assessments capture adolescents' stable beliefs and evaluative judgments about their relationships, providing a measure of global perceptions that extends beyond a simple aggregation of momentary states (Robinson & Clore, 2002). To align with the conceptualization of sensitization as a pre‐existing context for future interactions, we maintained temporal separation between general perceptions (assessed at baseline) and daily experiences (assessed across 21 days) (Chiang et al., 2023; Sloan et al., 2022).

For daily experiences, our goal was to capture the relational dynamics occurring in everyday life. We recognized that conflict and connectedness are dyadic relational processes wherein both adolescents' and parents' perspectives are valid and informative. Also, adolescents and parents may have divergent perspectives about their relationships, requiring us to test robustness across informants. The advantage of testing the robustness of findings across reporters rules out concerns about mono‐reporter bias, lending greater confidence in the findings. Therefore, we collected daily reports from both adolescents and parents to more fully capture actual daily relational experiences and reduce potential mono‐reporter bias. Specifically, our primary analyses relied on adolescent‐reported daily relationships to remain consistent with our theoretical focus on adolescent reactivity. We then incorporated parent‐reported daily relations as predictors of adolescent mood as robustness tests, allowing us to evaluate whether findings were robust and replicated across informants' perspectives on the same daily interactions.

Guided by the sensitization hypothesis (Cummings & Davies, 1994; Goeke‐Morey et al., 2013), we hypothesized that adolescents who perceive generally conflictual relationships with their parents would exhibit greater emotional reactivity to daily parent‐adolescent conflicts and variability in feelings of connectedness to their parents. On the other hand, we also hypothesized that adolescents who perceive generally close and connected relationships with their parents would exhibit less pronounced emotional reactions to daily variability in parent‐adolescent conflicts or connectedness.

## METHOD

Data were derived from the Penn State Family Life Optimizing Well‐being (FLOW) study, undertaken in high schools across central Pennsylvania from 2014 to 2017. Prior work using this dataset has documented key daily family processes and variability in adolescent moods. Daily parent‐adolescent conflict has been linked to greater daily adolescent angry mood and feeling less loved, whereas daily connectedness has been associated with higher daily adolescent subjective well‐being (Fosco, Brinberg, et al., 2021; LoBraico et al., 2020). Daily fluctuations in family conflict and cohesion have corresponded with daily adolescent mood and well‐being (Fosco & Lydon‐Staley, 2020). Previous research has also examined how general interparental conflict shapes adolescents' responses to daily interparental conflict (Sloan et al., 2022). The present study extends this body of work by examining how general parent‐adolescent relations contextualize daily parent‐adolescent conflict and connectedness, which have not been examined in prior publications. This study was approved by the Penn State's IRB (Protocol number: 0472, Title: Family Relationships and Adolescent WellBeing).

### Participants

Participants were parents and their adolescent children enrolled in Grade 9 or 10 from 150 families. Eligibility for participation was contingent upon several criteria: (a) two‐parent household status, (b) adolescents maintaining a single residence throughout the study period, (c) availability of internet access and the capacity to complete daily surveys from home, (d) proficiency in English, (e) the participating adolescent being in the 9th or 10th grade at the beginning of the study, and (f) mutual consent for participation from both the parent and adolescent involved.

Adolescent participants in this study were 92 females (61.3%) and 58 males (38.7%) (*M*_age_ = 14.60, *SD*_age_ = 0.83, range = 13–16). They were identified via parent report as White (83.3%), African American/Black (4.7%), Asian (4.7%), Native American/American Indian (0.7%), Hispanic/Latinx (0.7%), and Multiracial (5.3%). Parent participants in this study were 143 females (95%) and 7 males (5%) (*M*_age_ = 43.4, *SD*_age_ = 6.9, range = 30–61). Most of them identified as the adolescents' mother (92.7%), stepmother (1.3%), aunt (0.7%), and foster mother (0.7%). The remaining participants identified as fathers (4.7%). Parents were primarily White (89.3%), Asian/Asian American (3.3%), Black/African American (2.7%), Multiracial (3.3%), Hispanic/Latino (1.3%), or Native American/American Indian (0.7%). All households included two‐parent families, the majority of which were married (89%). Parents had been living together for an average of 18 years (SD = 7.2). With a median income of $70,000–$79,999 per year, annual household income varied from “less than $10,000” to “$125,000 or more”.

### Procedures

This study included a baseline survey and a 21‐day daily diary protocol, sent to the parent and the adolescent. Recruitment of parents was initially facilitated through emails distributed by school principals, followed by a snowballing approach via parent referrals. These communications included a link to a website containing research materials and a preliminary eligibility screener. Upon confirmation of a family's eligibility, parents and adolescents provided their consent to participate, completing baseline surveys thereafter. Subsequently, each night at 7:00 p.m., for 21 consecutive days, daily surveys were sent individually to parents and adolescents via email. To ensure timely completion, reminders were sent either through text messages or phone calls, according to the participants' preferred method. Although the survey links remained active until 9:00 a.m. the following morning, participants were encouraged to complete the surveys before bedtime. If the survey was completed in the morning, participants were instructed to report on their prior day's experiences. Participants were asked to complete surveys privately and independently for all surveys. Participants were compensated with a $25 Amazon or Walmart gift card for completing baseline surveys and up to $25 per week of daily surveys, depending on the number of daily surveys they completed.

For daily surveys, adolescents completed 90.5% (*M* = 19.00, *SD* = 2.52) and parents completed 96.5% (*M* = 20.27, *SD* = 1.28). Given the high compliance rate, all participants were included in analyses using all available daily data (days with missing data were excluded). To evaluate whether the number of daily diary reports completed was associated with key study variables, we examined correlations between report completion and both baseline and daily measures (Table S1). Among adolescents, greater diary completion was modestly associated with lower levels of daily parent‐adolescent conflict (both youth‐reported, *r* =−.22, *p* < .01, and parent‐reported, *r* = −.26, *p* < .01), lower negative affect (*r* = −.31, *p* < .01), higher positive affect (*r* = .24, *p* < .01), and lower baseline conflict (*r* = −.21, *p* < .01). For parents, greater diary completion was associated with lower levels of daily negative affect (*r* = −.18, *p* < .05) and parent‐reported conflict (*r* = −.20, *p* < .05). No other significant associations emerged.

### Measures

#### Daily measures

Both adolescents and parents reported their perceptions of these relationship experiences, while adolescents also reported their daily positive and negative affect. We calculated reliability scores for any measures that consisted of 2 or more items using indices appropriate for intensive longitudinal data, including within‐person variability ($R_{c}$; Bolger & Laurenceau, 2013) and between‐person reliability while accounting for repeated measures ($R_{1F}$; Cranford et al., 2006). Daily surveys were piloted among ten families to evaluate reliability and to determine whether the scales evidenced reliable within‐person change across 21 days.

##### Daily parent‐adolescent conflict

In the daily survey, adolescents and their parents each reported their perception of conflict with the other (Fosco et al., 2025; LoBraico et al., 2020), yielding adolescent‐reported conflict and parent‐reported conflict, respectively. Adolescents' daily feelings of conflict were assessed using two items, “How ANGRY or MAD was your [parent] with you,” and “How much TENSION was there between you and your [parent].” The text was customized to precisely indicate the caregiver's specific relationship to the adolescent (e.g., “mother”, “stepmother”). In parallel, parents reported their daily feelings of conflict with their child using the same two items, phrased as “I was ANGRY or MAD at my [child]” and “There was TENSION between my [child] and I today.” Daily conflict, computed separately for adolescents and parents as averages of the two items, ranged from not at all true (*0*) to very true (*10*), with higher scores indicating greater perceived conflict. These items assess parent‐adolescent emotional intensity of conflict, a core facet of conflict that reflects the degree of emotional negativity of interaction (Laursen et al., 1998). Emotional intensity of parent‐adolescent conflict is particularly salient during adolescence and is more developmentally important than conflict frequency (Laursen et al., 1998). In this sample, both adolescent‐ and parent‐reported conflict measures exhibited reliable within‐person and between‐person reliability across 21 days (Adolescents: $R_{C}\;$= .78, $R_{1F}$= .77; Parents: $R_{C}$= .72, $R_{1F}$= .58).

##### Daily parent‐adolescent connectedness

In the daily survey, adolescents and their parents each reported their perception of connectedness with the other, using a slider scale of 0 to 10 (in 0.1 increments) (Fosco, Brinberg, & Ram, 2021). Adolescents' daily feelings of connectedness were assessed using four items, “How close and connected did you feel to your [parent]?” “How warm and affectionate was your [parent] with you?” “How much did you feel loved by your [parent]?” and “How much did your [parent] care about your feelings?” The text was tailored to precisely reflect the caregiver's specific relationship to the adolescent (e.g., “mother”). In parallel, parents reported their daily feelings of connectedness using the same items, phrased as “I felt close and connected to my [child],” “I felt loved by my [child] today,” “I tried to understand my [child's] point of view,” and “I was loving and affectionate with my [child].” Daily connectedness was computed separately for adolescents and parents by averaging the four items, with higher scores indicating stronger perceived connectedness. In this sample, both adolescent‐ and parent‐reported connectedness measures demonstrated good within‐person and between‐person reliability across 21 days (Adolescents: $R_{C}$= .89, $R_{1F}$= .95; Parents: $R_{C}$= .80, $R_{1F}$= .90).

##### Daily adolescent negative and positive mood

In the daily survey, adolescents reported their negative and positive mood, with items selected from the Profile of Mood States (Curran et al., 1995). Adolescents’ daily negative mood was assessed using six items, each rated with the stem “How much of the time today did you feel…” with two items each for angry mood (angry; annoyed), depressed mood (depressed; sad or blue), anxious mood (worried; afraid). Responses to the six items were averaged to create a daily negative mood score, with a higher score reflecting greater negative mood. Daily positive mood was measured using two items: “How much of the time today did you feel happy?” and “How much of the time today did you feel content?” These two items were averaged to form daily positive mood, with a higher score reflecting greater positive mood. Adolescents rated their mood on a 10‐point scale that ranged from none of the time (*0*) to all of the time (*10*), with the option to adjust in 0.1 increments. All measures exhibited reliable within‐person and good between‐person reliability across 21 days for negative mood ($R_{C}$= .81, $R_{1F}$= .90) and positive mood ($R_{C}$= .80, $R_{1F}$= .87).

#### General measures

##### General adolescent‐reported conflict

In the baseline questionnaire, adolescents responded to five items capturing a general parent‐adolescent conflict from the Behavioral Affective Rating Scale (BARS; Spoth et al., 1998). Example items are as follows: “During the past month, how often did your [parent] get angry at you,” “how often did your [parent] shout, yell or scream at you.” Text was adapted to correctly identify each caregiver's role relative to the adolescent (e.g., “mother”). Items were rated on a 5‐point Likert scale from 1 (almost never) to 5 (almost always). Calculated average score of the items, with higher scores indicating higher levels of general parent‐adolescent conflict. This scale exhibited good reliability (Cronbach's *α* = .85).

##### General adolescent‐reported connectedness

In the baseline questionnaire, adolescents responded to 10 items capturing adolescents' general perception of connectedness with their parents from the Inventory of Parent and Peer Attachment (Armsden & Greenberg, 1989). Example items are as follows: “My [parent] trusts my judgment,” “When we discuss things, my [parent] considers my point of view,” and “My [parent] encourages me to talk about my difficulties.” Text was modified to reflect each caregiver's role relative to the adolescent (e.g., “mother”). Items were rated on a 5‐point Likert scale from 1 (completely untrue) to 5 (completely true). The calculated average score of the 10 items was used. Higher scores reflected higher general feelings of closeness with parents. This scale exhibited good reliability (Cronbach's *α* = .91).

### Analytic plan

We used multilevel models to capture daily adolescent negative mood and positive mood because these models are particularly well‐suited for addressing the inherent nested nature of intensive repeated measures – specifically, 21 days of observations nested within individuals (Bolger & Laurenceau, 2013). An important benefit of these models is their capacity to articulate dynamic (within‐person) facets of parent‐adolescent relationships and adolescents' moods (Bolger & Laurenceau, 2013). Before conducting the analyses, we computed the within‐person fluctuations in daily parent‐adolescent conflict and connectedness. Specifically, we person‐mean‐centered the conflict and connectedness scores by subtracting each individual's mean for each variable across all daily reports (i.e., the person mean) from their score on each daily occasion. These scores represent the deviation of conflict or connectedness on a specific day compared to an individual's typical daily level. Thus, positive values reflect days in which variables were higher than average for a particular individual. Then, we grand‐mean‐centered general variables (i.e., parent‐adolescent conflict and connectedness), except binary variables (e.g., sex), such that values reflect the degree to which an individual's general score compares to that of other individuals in the sample.

In the primary analysis, we ran two sets of multilevel models to understand the impact of daily conflict and connectedness on adolescent mood in a context of general parent‐adolescent conflict or connectedness, with days nested within individuals. We controlled for adolescent sex (female = 1), day of study (centered at the midpoint of the study; 0 = Day 10.5), and global level of the construct. To test our hypotheses, we ran two sets of multilevel models. The first set examined whether general parent‐adolescent conflict moderated the effects of daily parent‐adolescent conflict and connectedness. The second set tested whether general parent‐adolescent connectedness served as a moderator of these daily processes. Each model included a specific interaction term to conserve power and reduce the risk of Type II error. We took *Day's Conflict X General Connectedness* as an example to show the equation:

At level 1 (day‐level variables), the equation was constructed as follows:

(1) $$\begin{array}{cc} {\text{Mood}}_{\mathrm{it}}\;=\; & \beta_{0i}+\beta_{1i}\mathrm{Da}y^{\prime }{\text{sConflict}}_{\mathrm{it}}+\beta_{2i}\mathrm{Da}y^{\prime }{\text{sConnectedness}}_{\mathrm{it}}\\ & +\beta_{3i}{\mathrm{Day}}_{\mathrm{it}}+e_{\mathrm{it}} \end{array}$$

where ${\text{Mood}}_{\mathrm{it}}$ represents the adolescent mood for individual *i* on day *t*; $\beta_{0i}$ indicates the expected adolescent mood in the middle of the study for an individual experiencing an average level of daily conflict and connectedness; $\beta_{1i}$ indicates the association between daily conflict and adolescent mood; $\beta_{2i}$ indicates the association between daily connectedness and adolescent mood; $\beta_{3i}$ indicates the effect of time in a study on daily adolescent mood. Finally, $e_{\mathrm{it}}$ are day‐specific residuals that were allowed to autocorrelate (AR1).

At the second level (individual‐level variables), person‐specific intercepts and slopes from the Level 1 model were articulated as follows:

(2) $$\beta_{0i}=\gamma_{00}+\gamma_{01}\text{GConnectednes}s_{i}+\gamma_{02}\text{GConflic}t_{i}+\gamma_{03}Sex_{i}+u_{0i}$$

(3) $$\beta_{1i}=\gamma_{10}+\gamma_{11}{\text{GConnectedness}}_{i}+u_{1i}$$

(4) $$\beta_{2i}=\gamma_{20}+u_{2i}$$

(5) $$\beta_{3i}=\gamma_{30}$$

Here, the γs are sample‐level parameters and the $u^{\prime }$s are residual individual differences. These equations illustrate the association between general connectedness, general conflict, and sex with adolescent mood, marked by $\gamma_{01}$, $\gamma_{02},\text{and}\;\gamma_{03}$ respectively. $\gamma_{11}$ represents the cross‐level interaction between general connectedness (Level 2) and daily conflict (Level 1). This term captures whether the strength of the within‐person association between daily conflict and adolescent mood varies as a function of adolescents' general perceptions of connectedness. $u_{0i}$ ‐ $u_{2i}$ represents the random effects.

### Robustness tests

We conducted parallel models of parent‐adolescent relationships so that we could evaluate adolescent‐report and parent‐report of daily conflict and connectedness in separate models to facilitate comparisons for the robustness of the findings across reporters.

### Post hoc analyses

In addition to the primary models and robustness tests, we conducted five sets of post hoc analyses to address additional methodological considerations and evaluate the consistency of our findings. First, because our sample consisted primarily of mother‐adolescent dyads, we conducted analyses excluding other caregivers. We did not observe substantive differences in findings when analyzing only mother‐adolescent dyads (see Table S2). Therefore, to acknowledge all participants' contributions, the main analyses include the full sample. Second, to address concerns about potential Type I error and to align with our research questions, we ran two comprehensive models separately for general conflict and general connectedness. Third, to ensure consistency across models, we applied a False Discovery Rate correction across all model parameters, including both outcomes (i.e., negative and positive mood). Fourth, we included prior‐day affect as a covariate to account for potential carry‐over or autoregressive effects. Fifth, we examined whether the observed effects remained robust after accounting for additional covariates. We included an indicator for school day (1 = school day; 0 = weekend) and within‐ and between‐person levels of family cohesion and conflict to address conceptual overlap between family‐level and dyad‐level relationship quality. At the individual level, we included adolescent age, adolescent baseline depressive symptoms, the number of daily diary reports completed by adolescents, and parent sex.

## RESULTS

### Descriptive results

Table 1 provides descriptive statistics for key variables: daily parent‐adolescent connection and conflict, adolescent's daily positive and negative mood, adolescent sex, and general parent‐adolescent connectedness and conflict. For the global parent‐adolescent relationship, higher general parent‐adolescent connectedness was positively associated with adolescent positive mood (*r* = .43, *p* < .01) and negatively associated with negative mood (*r* = −.36, *p* < .01). In contrast, higher general parent‐adolescent conflict was negatively associated with adolescent positive mood (*r* = −.29, *p* < .01) and positively associated with negative mood (*r* = .46, *p* < .01). We also examined the between‐person level correlations (i.e., average levels across study days) among the daily variables. Parent‐reported measures were moderately correlated with adolescent‐reported connectedness and conflict (connectedness: *r* = .37, *p* < .01; conflict: *r* = .49, *p* < .01). Positive mood and negative mood were strongly negatively correlated at the between‐person level (*r* = −.63, *p* < .01).

**TABLE 1:** Descriptive statistics and correlation matrix of key variables.

Variable	1	2	3	4	5	6	7	8	9
1. Day's connect (A)	‐‐	−.58**	.37**	−.31**	.62**	−.38**	.05**	.60**	−.38**
2. Day's conflict (A)	−.43**	‐‐	−.22**	.49**	−.46**	.68**	−.10**	−.43**	.57**
3. Day's connect (P)	.30**	−.23**	‐‐	−.51**	.26**	−.26**	.19**	.21**	−.13**
4. Day's conflict (P)	−.22**	.34**	−.34**	‐‐	−.14**	.33*	−.19**	−.19**	.28**
5. Day's positive mood	.27**	−.20**	.16**	−.14**	‐‐	−.63**	−.10**	.43**	−.29**
6. Day's negative mood	−.24**	.28**	−.13**	.12**	−.50**	‐‐	.13**	−.36**	.46**
7. Adolescent sex	‐‐	‐‐	‐‐	‐‐	‐‐	‐‐	‐‐	.02	.01
8. General connect (A)	‐‐	‐‐	‐‐	‐‐	‐‐	‐‐	‐‐	‐‐	−.40**
9. General conflict (A)	‐‐	‐‐	‐‐	‐‐	‐‐	‐‐	‐‐	‐‐	‐‐
*M*	8.43	1.03	8.05	0.96	8.11	1.34	‐‐	4.23	1.66
*SD*	1.78	1.36	1.64	1.03	2.26	1.92	‐‐	0.68	0.69
*ICC*	67.21%	42.92%	65.18%	27.76%	57.31%	62.04%	‐‐	‐‐	‐‐

*Note*: Between‐person (average) correlations are shown above the diagonal; within‐person (daily) correlations are shown below the diagonal.

Abbreviations: A, adolescent‐report; Connect, Connectedness; ICC, intraclass correlation coefficient; M, mean; P, parent‐report; SD, standard deviation.

* *p* < .05.

** *p* < .01.

Notably, intraclass correlations (ICC) of daily variables representing person‐level clustering ranged from 27.76% (for parent‐reported parent‐adolescent conflict) to 67.21% (for adolescent‐reported parent‐adolescent connectedness). ICC indicated that 27.76% to 67.21% of the variability in these daily variables is attributable to between‐person differences, with the remaining variability reflecting meaningful within‐person fluctuations over time. These ICC values exceed the commonly accepted threshold (i.e., <80% ICC) for demonstrating meaningful within‐person variability, supporting the appropriateness of daily‐level analyses (Bolger & Laurenceau, 2013). At the within‐person level, daily connectedness was positively associated with positive mood (*r* = .27, *p* < .01 for adolescents; *r* = .16, *p* < .01 for parents) and negatively associated with negative mood (*r* = −.24, *p* < .01 for adolescents; *r* = −.13, *p* < .01 for parents). Daily conflict was associated with more negative mood (*r* = .28, *p* < .01 for adolescents; *r* = .12, *p* < .01 for parents) and less positive mood (*r* = −.20, *p* < .01 for adolescents; *r* = −.14, *p* < .01 for parents).

### Primary analysis: Adolescent‐report models

First, results indicated the significant main effects of daily parent‐adolescent connectedness and conflict on adolescent daily moods. Daily parent‐adolescent connectedness was consistently associated with better mood, indicating higher positive mood (*b* = .30, *p* < .01) and lower negative mood (*b* = −.15 to −.14, *p* < .01). In contrast, daily parent‐adolescent conflict was associated with worse mood, resulting in higher negative mood (*b* = .11 to .13, *p* < .01) and lower positive mood (*b* = −.09 to −.08, *p* < .01). Beyond these daily associations, general parent‐adolescent relationship quality was found to be significantly linked to adolescent moods. Higher general connectedness was linked with higher positive mood (*b* = 1.02 to 1.06, *p* < .01) and lower negative mood (*b* = −.54 to −.52, *p* < .01), while the general conflict was associated with higher negative moods (*b* = .80 to .82, *p* < .01) but not associated with positive moods.

**FIGURE 2:**
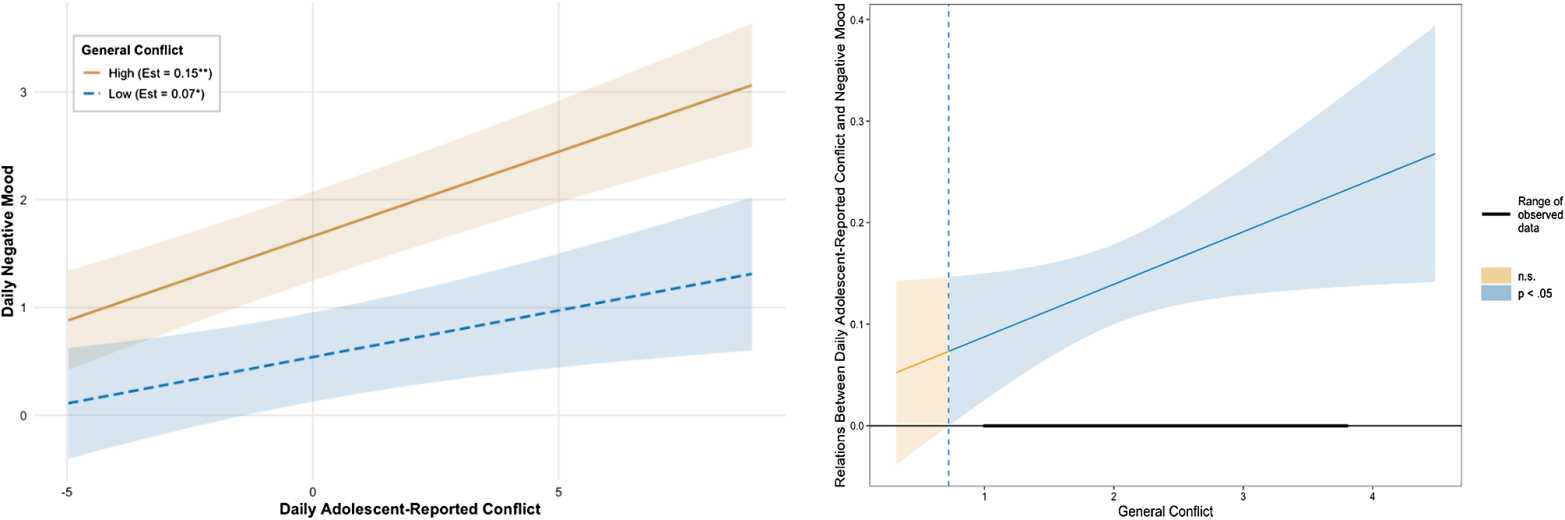
Role of general parent‐adolescent conflict. Low and high values of the general parent‐adolescent conflict represent −1 SD below and + 1 SD above the mean, respectively.

**FIGURE 3:**
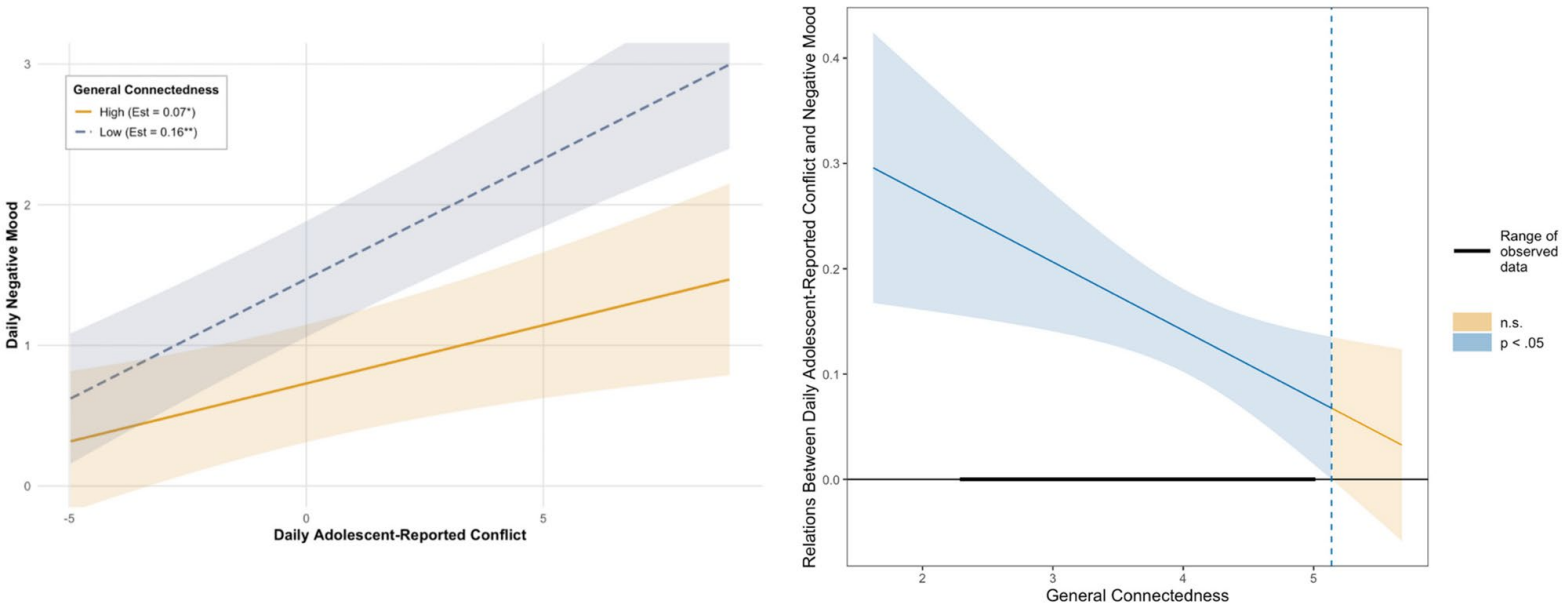
Role of general parent‐adolescent connectedness. Low and high values of the general parent‐adolescent connectedness represent −1 SD below and + 1 SD above the mean, respectively.

Turning to the interaction effects, as shown in Table 2, general parent‐adolescent conflict moderated the association between daily fluctuations in parent‐adolescent conflict and adolescent negative mood (*b* = .05, *p* < .05). We probed significant interactions using simple slope analyses and the Johnson‐Neyman technique. For all significant cross‐level interactions, simple slopes were estimated at low (−1 SD below the mean) and high (+1 SD above the mean) values of the moderator. As depicted in Figure 2, when the general conflict was higher, adolescents exhibited stronger linkages between daily parent‐adolescent conflict and daily negative mood (low general conflict: *b* = 0.07, *p* < .05; high general conflict: *b* = 0.15, *p* < .01). As shown in Table 3, general parent‐adolescent connectedness moderated the daily positive linkages between parent‐adolescent conflict and adolescent negative mood (*b* = −.07, *p* < .05). As depicted in Figure 3, the positive association between daily parent‐adolescent conflict and daily negative mood was weaker when general connectedness was higher, suggesting a buffering effect (low general connectedness: *b* = 0.16, *p* < .01; high general connectedness: *b* = 0.07, *p* < .05). No significant results were found regarding adolescent positive mood to support the moderating role of general parent‐adolescent conflict or connectedness. The effect of daily parent‐adolescent connectedness was not moderated by general parent‐adolescent relationship characteristics (i.e., conflict and connectedness).

**TABLE 2:** Testing the moderating role of general parent‐adolescent conflict – adolescent models.

	Adolescent‐report			
	Negative mood		Positive mood	
Fixed effects	*Est. (SE)*	*Est. (SE)*	*Est. (SE)*	*Est. (SE)*
Intercept	1.12 (0.18) **	1.12 (0.18) **	8.35 (0.21) **	8.35 (0.21) **
Day's connect	−0.15 (0.03) **	−0.15 (0.03) **	0.30 (0.04) **	0.30 (0.04) **
Day's conflict	0.11 (0.02) **	0.13 (0.02) **	−0.08 (0.02) **	−0.08 (0.02) **
Day of study	−0.02 (0.00) **	−0.02 (0.00) **	0.01 (0.01)	0.01 (0.01)
Adol sex	0.45 (0.23) *	0.46 (0.23) *	−0.47 (0.26)	−0.47 (0.26)
GConnect	−0.52 (0.18) **	−0.52 (0.18) **	1.06 (0.20) **	1.06 (0.20) **
GConflict	0.82 (0.17) **	0.81 (0.17) **	−0.28 (0.20)	−0.28 (0.20)
Day's conflict * GConflict	0.05 (0.03) *	‐‐	0.00 (0.03)	‐‐
Day's connect * GConflict	‐‐	−0.02 (0.04)	‐‐	0.02 (0.05)
Random effects				
Intercept	1.75 (1.32)	1.75 (1.32)	2.33 (1.53)	2.33 (1.53)
Day's connect	0.05 (0.22)	0.05 (0.22)	0.06 (0.24)	0.06 (0.25)
Day's conflict	0.01 (0.10)	0.01 (0.11)	0.00 (0.07)	0.00 (0.07)
Residual	1.23 (1.11)	1.23 (1.11)	2.00 (1.41)	2.00 (1.41)

Abbreviations: Adol Sex, Adolescent sex; Day's Conflict, Daily P‐A conflict; Day's Connect, Daily P‐A connectedness; Est., Estimate; GConflict, General P‐A conflict; GConnect, General P‐A connectedness; SE, Standard Error.

* *p* < .05.

** *p* < .01.

**TABLE 3:** Testing the moderating role of general parent‐adolescent connectedness – adolescent models.

	Adolescent‐report			
	Negative mood	Positive mood		
Fixed effects	*Est. (SE)*	*Est. (SE)*	*Est. (SE)*	*Est. (SE)*
Intercept	1.12 (0.18) **	1.12 (0.18) **	8.35 (0.21) **	8.35 (0.21) **
Day's connect	−0.15 (0.03) **	−0.14 (0.03) **	0.30 (0.04) **	0.30 (0.04) **
Day's conflict	0.11 (0.02) **	0.13 (0.02) **	−0.09 (0.02) **	−0.08 (0.02) **
Day of study	−0.02 (0.00) **	−0.02 (0.00) **	0.01 (0.01)	0.01 (0.01)
Adol sex	0.45 (0.23) **	0.45 (0.23) **	−0.48 (0.26)	−0.47 (0.26)
GConnect	−0.54 (0.18) **	−0.53 (0.18) **	1.02 (0.20) **	1.06 (0.20) **
GConflict	0.80 (0.17) **	0.81 (0.17) **	−0.29 (0.20)	−0.28 (0.20)
Day's conflict * GConnect	−0.07 (0.03) **	‐‐	−0.04 (0.03)	‐‐
Day's connect * GConnect	‐‐	0.06 (0.05)	‐‐	0.01 (0.06)
Random effects				
Intercept	1.75 (1.32)	1.75 (1.32)	2.34 (1.53)	2.33 (1.53)
Daily connect	0.05 (0.22)	0.05 (0.22)	0.06 (0.25)	0.06 (0.25)
Daily conflict	0.01 (0.10)	0.01 (0.11)	0.00 (0.06)	0.00 (0.06)
Residual	1.23 (1.11)	1.23 (1.11)	2.00 (1.41)	2.00 (1.41)

Abbreviations: Adol Sex, Adolescent sex; Day's Conflict, Daily P‐A conflict; Day's Connect, Daily P‐A connectedness; Est., Estimate; GConflict, General P‐A conflict; GConnect, General P‐A connectedness; SE, Standard Error.

* *p* < .05.

** *p* < .01.

### Robustness tests: Parent‐report models

To examine the robustness of our findings, we sought to replicate findings from the adolescent‐report models in analyses using parent‐reported daily parent‐adolescent connectedness and conflict as predictors of adolescent mood. As shown in Table 4, results from these parallel parent‐report models largely mirrored the primary adolescent‐report findings. Consistent with adolescent‐report models, general parent‐adolescent conflict or connectedness moderated the daily linkage between conflict and adolescent negative mood. Specifically, the positive association between daily parent‐adolescent conflict and adolescent negative mood became stronger when general parent‐adolescent conflict was higher (*b* = .06, *p* < .05) or general parent‐adolescent connectedness was lower (*b* = −.05, *p* < .05). Overall, these robustness tests strengthened confidence in the robustness of our primary interaction findings across different reporters of daily parent‐adolescent relations.

**TABLE 4:** Testing the moderating role of general parent‐adolescent relationships – parent models.

	Negative mood				Positive mood			
	Est. (SE)	Est. (SE)	Est. (SE)	Est. (SE)	Est. (SE)	Est. (SE)	Est. (SE)	Est. (SE)
Fixed effects								
Intercept	1.11 (0.18) **	1.10 (0.18) **	1.11 (0.18) **	1.11 (0.18) **	8.30 (0.20) **	8.30 (0.20) **	8.30 (0.20) **	8.30 (0.20) **
Day's connect	−0.11 (0.03) **	−0.11 (0.03) **	−0.11 (0.03) **	−0.11 (0.03) **	0.16 (0.03) **	0.16 (0.03) **	0.16 (0.03) **	0.16 (0.03) **
Day's conflict	0.05 (0.02) *	0.06 (0.02) **	0.05 (0.02) **	0.06 (0.02) **	−0.09 (0.03) **	−0.09 (0.03) **	−0.09 (0.03) **	−0.09 (0.03) **
Day of study	−0.02 (0.00) **	−0.02 (0.00) **	−0.02 (0.00) **	−0.02 (0.00) **	0.01 (0.01)	0.01 (0.01)	0.01 (0.01)	0.01 (0.01)
Adol sex	0.48 (0.22) *	0.48 (0.23) *	0.48 (0.22) *	0.48 (0.22) *	−0.40 (0.26)	−0.40 (0.26)	−0.40 (0.26)	−0.40 (0.26)
GConnect	−0.50 (0.17) **	−0.50 (0.17) **	−0.49 (0.17) **	−0.55 (0.18) **	1.00 (0.20) **	1.00 (0.20) **	1.01 (0.20) **	1.02 (0.20) **
GConflict	0.82 (0.17) **	0.85 (0.17) **	0.83 (0.17) **	0.83 (0.17) **	−0.33 (0.20)	−0.30 (0.20)	−0.31 (0.20)	−0.31 (0.20)
Day's conflict * GConflict	0.06 (0.03) *	‐‐	‐‐	‐‐	−0.05 (0.04)	‐‐	‐‐	‐‐
Day's connect * GConflict	‐‐	−0.04 (0.04)	‐‐	‐‐	‐‐	−0.02 (0.05)	‐‐	‐‐
Day's conflict * GConnect	‐‐	‐‐	−0.05 (0.02) *	‐‐	‐‐	‐‐	0.01 (0.03)	‐‐
Day's connect * GConnect	‐‐	‐‐	‐‐	0.07 (0.04)	‐‐	‐‐	‐‐	−0.02 (0.05)
Random effects								
Intercept	1.75 (1.32)	1.75 (1.32)	1.75 (1.32)	1.75 (1.32)	2.30 (1.52)	2.30 (1.52)	2.30 (1.52)	2.30 (1.52)
Day's connect	0.03 (0.18)	0.03 (0.18)	0.03 (0.18)	0.03 (0.18)	0.05 (0.22)	0.05 (0.22)	0.05 (0.22)	0.05 (0.22)
Day's conflict	0.01 (0.08)	0.01 (0.08)	0.01 (0.08)	0.01 (0.08)	0.02 (0.14)	0.02 (0.14)	0.02 (0.14)	0.02 (0.14)
Residual	1.37 (1.17)	1.37 (1.17)	1.37 (1.17)	1.37 (1.17)	2.09 (1.45)	2.09 (1.45)	2.09 (1.45)	2.09 (1.45)

Abbreviations: Adol Sex, Adolescent sex; Day's Conflict, Daily P‐A conflict; Day's Connect, Daily P‐A connectedness; Est., Estimate; GConflict, General P‐A conflict; GConnect, General P‐A connectedness; SE, Standard Error.

* *p* < .05.

** *p* < .01.

### Post hoc analyses

All supplementary post hoc results are presented in Tables S2–S8. Post hoc analyses revealed no substantive differences when (1) restricting the sample to mother‐adolescent dyads only, (2) examining the comprehensive models for general conflict or connectedness, or (3) including additional covariates (i.e., within‐ and between‐person levels of family cohesion and conflict, school days, number of completed adolescent diaries, adolescent age, adolescent baseline depressive symptom, and parent sex). After FDR correction across all interaction parameters (Table S5) and after additionally controlling for prior‐day mood (Table S6), the moderating role of general parent‐adolescent connectedness remained statistically significant (FDR‐adjusted *p* = .016; with prior‐day control *p* = .019), whereas the moderating role of general parent‐adolescent conflict was no longer statistically significant (FDR‐adjusted *p* = .055; with prior‐day control *p* = .076).

## DISCUSSION

This study deepens our understanding of parent‐adolescent conflict and connectedness by examining whether daily processes are shaped by general parent‐adolescent relationships. In a preliminary assessment using reports from both parents and adolescents, our results indicate that parent‐adolescent conflict and connectedness exhibit meaningful day‐to‐day fluctuations. Also, we replicate previous studies (Chiang et al., 2023; Fosco, Brinberg, & Ram, 2021), indicating that daily parent‐adolescent relationships correspond to daily variability in adolescents' moods; specifically, on days when parent‐adolescent conflict was lower or connectedness was higher than usual, adolescents reported higher positive mood and lower negative moods. Beyond the daily ebb and flow of parent‐adolescent relationship processes, our results suggest that the general levels of parent‐adolescent conflict and connectedness also play a critical role in shaping daily linkages between conflict and negative mood. By simultaneously considering daily and general levels of parent‐adolescent relationships, our study expands traditional views, distinguishing processes that unfold across different timescales.

### General conflict as a sensitization context of daily parent‐adolescent conflict

Our first hypothesis – guided by the sensitization perspective – proposed that the association between daily relationships and adolescent mood would be stronger at higher levels of general parent‐adolescent conflict. This hypothesis was partially supported: general parent‐adolescent conflict served as a moderator only in the context of daily conflict. As expected, for adolescents who experienced higher levels of general parent‐adolescent conflict, daily parent‐adolescent conflict was more strongly related to higher daily negative mood, compared to adolescents who experienced lower general parent‐adolescent conflict. Our study benefitted from both parent and adolescent reports of daily parent‐adolescent relationships, which helped address the limitation of mono‐reporter bias; thus, findings were robust across reporters. These results align with the sensitization hypothesis and existing research, which suggest that adolescents with a history of high conflict exhibit greater reactivity to future conflicts compared to their peers (e.g., Davies et al., 2006; Sloan et al., 2022). Our findings also extend the sensitization perspective to parent‐adolescent conflict. Rather than habituating, higher levels of general parent‐adolescent conflicts lower adolescents' threshold for emotional reactivity and lead them to expect and react strongly to conflict in daily interactions. Qualitative research further supports this process: adolescents from high‐conflict families describe feeling “oppressed” and report that repeated conflicts lead them to expect dismissal of their perspectives, heightening emotional responses to daily conflict (Nguyen & Nguyen, 2023).

Interestingly, the sensitization hypothesis was supported in the context of daily parent‐adolescent conflict but not daily parent‐adolescent connectedness. General levels of parent‐adolescent conflict did not moderate reactivity to daily connectedness. Drawing from sensitization research on interparental conflict, adolescents exposed to high levels of general conflict tend to perceive greater threat and self‐blame from daily interparental conflict (Sloan et al., 2022), which may mediate the link between daily conflict and mood. It is possible that daily parent‐adolescent conflict similarly activates these appraisal processes, whereas daily connectedness– as a positive experience–may not engage the same pathways. Future research should examine lagged processes to determine whether negative appraisals mediate these daily effects. Notably, daily connectedness consistently emerged as a protective factor, even in the context of high general conflict.

### General connectedness as a buffering context of daily parent‐adolescent conflict

Our second hypothesis, that higher global parent‐adolescent connectedness would mitigate the link between daily parent‐adolescent relationships and adolescents' moods, was also partially supported. General parent‐adolescent connectedness only moderated the association between daily parent‐adolescent conflict and negative mood. As expected, for parent‐adolescent relationships characterized by high general connectedness, daily conflict was less strongly associated with adolescents' negative moods. Results were consistent across adolescent‐ and parent‐reported daily measures, underscoring the robustness of these findings across reporters. These findings are consistent with prior studies that support the buffering role of positive parent‐adolescent relationships in the negative effects of daily negative events (Lippold et al., 2016; Vannucci et al., 2019).

Our findings highlight the distinct moderating roles of general parent‐adolescent conflict and connectedness. Conceptually, these constructs represent related but separable dimensions of relationship quality that appear to operate through different psychological mechanisms in shaping adolescents' daily emotional experiences (Forgatch & DeGarmo, 1998). General conflict may heighten adolescents' sensitivity to negative interactions, lowering their threshold for emotional reactivity in daily life. In contrast, general connectedness may function as a protective resource rooted in attachment theory, whereby adolescents who maintain stable, trusting, and close relationships with their parents develop a sense of security in their daily lives (Bowlby, 1988; Moretti & Peled, 2004). Adolescents with high general connectedness know they have a supportive and secure relationship to fall back on (McElhaney et al., 2009; McKenna et al., 2022), thereby perceiving daily conflicts as less reflective of problems in their relationship with their parents, and thus experiencing conflicts as less distressing. Furthermore, our results support that high general parent‐adolescent connectedness allows them to maintain lower baseline levels of negative mood, indicating that this secure foundation helps adolescents develop better emotional regulation skills (Kerns et al., 2015). Connected parents facilitate this emotional regulation by offering problem‐solving assistance, providing guidance on managing negative events, and serving as models of self‐regulation, which collectively help diminish adolescents' emotional responses to daily conflict (Lippold et al., 2016).

It is interesting to note that general parent‐adolescent connectedness did not moderate the within‐person associations between connectedness and moods. In other words, adolescents benefit from feeling connected with their parents on a given day ‐ reporting lower negative mood ‐ regardless of their overall level of connectedness. One possible explanation is that daily experiences of connectedness fulfill adolescents' universal psychological need for relatedness and a sense of security (Bowlby, 1988; Deci & Ryan, 2000), resulting in consistent emotional benefits even in families with lower overall connectedness. These robust within‐person associations support the short‐term resilience model (Bai & Repetti, 2015), in which episodes of connectedness may foster adolescents' resilience and emotion regulation skills and, over time, accumulate to promote long‐term resilience. Consistent with prior research, daily fluctuations in parent‐adolescent connectedness play a significant role in shaping long‐term adolescent outcomes, including depression, anxiety, antisocial behavior, and substance use (Fosco et al., 2019; Fosco & LoBraico, 2019).

Interestingly, adolescents' daily positive mood was related to daily parent‐adolescent conflict and connectedness, regardless of general relationships. A possible explanation is that positive mood is more sensitive to concurrent relationship experiences, and day‐to‐day relationship experiences, as evidenced by lower between‐person variability (i.e., lower ICC). This is supported by our results showing that general conflict was not associated with positive mood, while daily conflict and connectedness were. These findings demonstrate the robust within‐person linkages between daily relationship experiences and adolescent daily positive mood (Fosco & Lydon‐Staley, 2020; Vannucci et al., 2019). Together, these results emphasize the need to differentiate between the predictors of positive and negative mood across different timescales. Future research integrating qualitative methods, such as interviews or open‐ended daily assessments, could provide deeper insight into the nuanced mechanisms impacting positive and negative moods.

### Implications

First, this study integrates general levels and daily parent‐adolescent relationships by using baseline and daily diary surveys. The findings highlight the importance of considering multiple timescales in understanding the links between parent‐adolescent relationships and adolescent mood. Second, general parent‐adolescent connectedness and conflict significantly shape the daily processes of parent‐adolescent conflict, highlighting the importance of addressing these trait‐like relational patterns. These findings underscore the importance of family‐focused intervention aimed at fostering generally positive parent‐adolescent relationship patterns, characterized by low conflict and high connectedness, which can reduce the negative impact of daily conflict on adolescent well‐being. Notably, the buffering role of general connectedness was particularly robust across post hoc analyses, suggesting that strengthening the broader closeness of the parent‐adolescent relationship may be an especially promising intervention target for helping adolescents navigate day‐to‐day conflicts. Third, the daily connectedness process operates independently of general relationships, suggesting that interventions should emphasize the reliable benefits of everyday parent‐adolescent connectedness and focus on fostering positive interactions in daily life. Fourth, daily adolescent positive mood is consistently shaped by daily parent‐adolescent interactions, regardless of general relationships. Thus, apart from general relationships, attention should be given to daily interactions between parents and adolescents to promote daily well‐being. Lastly, both daily conflict and connectedness matter in daily life and make unique contributions to daily mood; therefore, interventions should address both aspects of the parent‐adolescent relationship.

### Limitations and further direction

Limitations and further directions should be considered in our study. First, future research would benefit from including more diverse families to better understand the generalizability of these findings across broader populations. This may include families from varied racial and socioeconomic backgrounds, as well as those with different structures ‐ such as families with multiple parents or adolescents who divide their time between two homes. Second, as most parent participants in our study were mothers, it is not clear whether the current findings generalize to father‐adolescent relationships. Further work is needed to investigate the role of father‐child conflict sensitization processes. Third, although the sensitization hypothesis implies a directional effect from the general relationship to adolescents' emotional reactivity to daily relations, daily processes are likely bidirectional. However, our daily data are cross‐sectional in nature. Future research using intensive or observational methods could clarify these immediate reciprocal dynamics. Fourth, although daily parent‐adolescent conflict demonstrated acceptable reliability for adolescent reports and acceptable within‐person reliability for parent reports, and has been used in prior studies (e.g., Fosco et al., 2025; LoBraico et al., 2020), we acknowledge that its between‐person reliability for parent reports was moderate. Future work using measures with stronger between‐person reliability would further strengthen confidence in these findings. Lastly, data were collected approximately a decade ago, and we recognize that contextual factors, such as increased smartphone and social media use, may have altered parent‐adolescent interactions. We recommend that future research replicate these findings with current samples to establish generalizability and identify which processes remain robust.

## CONCLUSION

This study examined how general parent‐adolescent contexts shaped adolescents' responses to daily parent‐adolescent conflict and connectedness. We applied multilevel modeling to simultaneously consider general levels and daily fluctuations in parent‐adolescent relationships, providing a more nuanced understanding of how these dynamics are associated with adolescent mood. The daily association between parent‐adolescent conflict and adolescents' negative mood was found to depend on general levels of conflict and connectedness. Specifically, our findings extend the sensitization hypothesis to parent‐adolescent conflict, indicating that adolescents' general histories of conflict with their parents sensitize them to daily experiences. Also, the results support attachment processes, suggesting that the positive association between daily conflict and adolescents' negative moods is weaker at higher levels of general parent‐adolescent connectedness. Notably, we identified a strong within‐person linkage between daily parent‐adolescent connectedness and adolescent mood, independent of the general relationship. In summary, our findings highlight that general and daily parent‐adolescent relationships work together to shape adolescents' well‐being. General relationship quality provides a meaningful context for understanding adolescents' emotional responses to daily conflict. At the same time, beyond these general patterns, adolescents benefit significantly from the day‐to‐day variability in their interactions with parents—particularly connectedness.

## SUPPLEMENTARY MATERIAL

The supplementary material includes correlations between the number of completed daily diary reports and key study variables, as well as five sets of post hoc analyses evaluating the robustness of the findings: mother‐adolescent dyad models, full models, FDR correction, prior‐day outcome models, and models with additional covariates.

## AUTHOR CONTRIBUTIONS

**Lan Chen:** Conceptualization; methodology; formal analysis; funding acquisition; visualization; software; writing – review and editing; writing – original draft. **Carlie J. Sloan:** Writing – review and editing; software; formal analysis. **Gregory M. Fosco:** Conceptualization; data curation; formal analysis; funding acquisition; investigation; methodology; project administration; resources; supervision; writing – review and editing. **Damon E. Jones:** Formal analysis; writing – review and editing.

## CONFLICT OF INTEREST STATEMENT

The authors declare that they have no conflict of interest.

## ETHICAL APPROVAL

Ethical approval for the FLOW study was granted by Penn State's IRB (Protocol number: 0472, Title: Family Relationships and Adolescent Well‐Being).

## FUNDING STATEMENT

Data collection was supported by the Karl R. and Diane Wendle Fink Early Career Professorship for the Study of Families and Social Sciences Research Institute, Pennsylvania State University (Fosco). Greg Fosco was supported by the Edna P. Bennett Faculty Fellowship in Prevention Research through the College of Health and Human Development, Pennsylvania State University. Carlie Sloan and Lan Chen were supported by the Prevention and Methodology Training Program (T32 DA017629; MPIs: J. Maggs & S. Lanza) with funding from the National Institute on Drug Abuse. Carlie Sloan was also supported by the National Institute on Drug Abuse T32 DA039772 (MPIs: N. Gonzales & C. Berkel). The content is solely the responsibility of the authors and does not necessarily represent the official views of the funding agencies.

## MATERIALS AND CODE AVAILABILITY

Materials and analytic code are available on request from the corresponding author.

## PATIENT CONSENT STATEMENT

Informed consent was obtained from both parents and adolescents for their participation in the study.

## Supporting information


**Table S1.** Correlations Between Number of Completed Daily Diary Reports and Key Study Variables
**Table S2**. Testing the Moderating Role of General Mother‐Adolescent Relationships
**Table S3**. Full Model for General Parent‐Adolescent Conflict
**Table S4**. Full Model for General Parent‐Adolescent Connectedness
**Table S5**. Results of False Discovery Rate (FDR) Correction for Cross‐Level Interactions
**Table S6**. Models Adding Prior‐Day Outcome
**Table S7**. Correlations Between Family‐Level and Parent‐Adolescent Relationships
**Table S8**. Models Testing Additional Covariates

## Data Availability

The data that support the findings of this study are available on request from the corresponding author. The data are not publicly available due to privacy or ethical restrictions.
