# Supplementary material for: Evaluating general parent‐adolescent relations as a context for daily relationship processes and adolescent mood
Source: J Res Adolesc. 2026 Jul 10;36(3):e70224. doi: 10.1111/jora.70224 (PMC13354831; doi:10.1111/jora.70224)
Supplement: Supplementary file 1 — Table S1. Correlations Between Number of Completed Daily Diary Reports and Key Study Variables Table S2. Testing the Moderating Role of General Mother‐Adolescent Relationships Table S3. Full Model for General Parent‐Adolescent Conflict Table S4. Full Model for General Parent‐Adolescent Connectedness Table S5. Results of False Discovery Rate (FDR) Correction for Cross‐Level Interactions Table S6. Models Adding Prior‐Day Outcome Table S7. Correlations Between Family‐Level and Parent‐Adolescent Relationships Table S8. Models Testing Additional Covariates [file JORA-36-0-s001.docx]

**Table S1.** Correlations Between Number of Completed Daily Diary Reports and Key Study Variables

| **Variable** | **Youth r** | **p-value** | **Parent r** | **p-value** |
| --- | --- | --- | --- | --- |
| Day’s Connectedness (A) | 0.08 | .343 | -0.10 | .224 |
| Day’s Conflict (A) | -0.22** | .007 | -0.06 | .466 |
| Day’s Positive Mood (A) | 0.24** | .003 | 0.05 | .561 |
| Day’s Negative Mood (A) | -0.31** | .000 | -0.18* | .028 |
| Day’s Connectedness (P) | 0.07 | .368 | -0.09 | .275 |
| Day’s Conflict (P) | -0.26** | .001 | -0.20* | .013 |
| General Connectedness (A) | 0.08 | .330 | 0.00 | .989 |
| General Conflict (A) | -0.21** | .009 | -0.04 | .621 |
| Adolescent Sex | 0.03 | .730 | -0.07 | .395 |
| Parent Sex | -0.04 | .592 | 0.06 | .460 |
| Family Income | -0.06 | .493 | 0.11 | .179 |

Note. A = adolescent-report, P = parent-report, r = Pearson correlation, ** p < .01, * p < .05

**Table S2.** Testing the Moderating Role of General Mother-Adolescent Relationships

|  | *Negative Mood* | | | | *Positive Mood* | | | |
| --- | --- | --- | --- | --- | --- | --- | --- | --- |
| Fixed Effects | *Est. (SE)* | *Est. (SE)* | *Est. (SE)* | *Est. (SE)* | *Est. (SE)* | *Est. (SE)* | *Est. (SE)* | *Est. (SE)* |
| Intercept | 1.15 (0.18) ** | 1.15 (0.18) ** | 1.15 (0.18) ** | 1.15 (0.18) ** | 8.31 (0.20) ** | 8.31 (0.20) ** | 8.31 (0.20) ** | 8.31 (0.20) ** |
| Day’s Connect | -0.14 (0.03) ** | -0.13 (0.03) ** | -0.14 (0.03) ** | -0.13 (0.03) ** | 0.30 (0.04) ** | 0.30 (0.04) ** | 0.30 (0.04) ** | 0.30 (0.04) ** |
| Day’s Conflict | 0.11 (0.02) ** | 0.13 (0.02) ** | 0.12 (0.02) ** | 0.13 (0.02) ** | -0.09 (0.02) ** | -0.08 (0.02) ** | -0.09 (0.02) ** | -0.08 (0.02) ** |
| Day of Study | -0.02 (0.00) ** | -0.02 (0.00) ** | -0.02 (0.00) ** | -0.02 (0.00) ** | 0.01 (0.01) * | 0.01 (0.01) * | 0.01 (0.01) * | 0.01 (0.01) * |
| Adol Sex | 0.40 (0.24) | 0.40 (0.24) | 0.40 (0.23) | 0.40 (0.24) | -0.35 (0.26) | -0.35 (0.26) | -0.36 (0.26) | -0.35 (0.26) |
| G­­Connect | -0.53 (0.18) ** | -0.54 (0.18) ** | -0.55 (0.18) ** | -0.54 (0.18) ** | 0.99 (0.20) ** | 0.99 (0.20) ** | 0.94 (0.20) ** | 0.99 (0.20) ** |
| GConflict | 0.83 (0.18) ** | 0.83 (0.18) ** | 0.82 (0.18) ** | 0.82 (0.18) ** | -0.34 (0.20) | -0.36 (0.20) | -0.37 (0.20) | -0.36 (0.20) |
| Day’s Conflict * GConflict | 0.06 (0.02) * | -- | -- | -- | 0.01 (0.03) | -- | -- | -- |
| Day’s Connect* GConflict | -- | -0.04 (0.04) | -- | -- | -- | 0.02 (0.05) | -- | -- |
| Day’s Conflict * GConnect | -- | -- | \| -0.06 (0.03) * \| \| --- \| | -- | -- | -- | -0.04 (0.03) | -- |
| Day’s Connect * GConnect | -- | -- | -- | 0.06 (0.05) | -- | -- | -- | 0.00 (0.06) |
| Random Effects |  |  |  |  |  |  |  |  |
| Intercept | 1.79 (1.34) | 1.79 (1.34) | 1.79 (1.34) | 1.79 (1.34) | 2.22 (1.49) | 2.22 (1.49) | 2.22 (1.49) | 2.22 (1.49) |
| Day’s Connect | 0.05 (0.22) | 0.05 (0.22) | 0.05 (0.22) | 0.05 (0.22) | 0.06 (0.25) | 0.06 (0.25) | 0.06 (0.25) | 0.06 (0.25) |
| Day’s Conflict | 0.01 (0.10) | 0.01 (0.11) | 0.01 (0.10) | 0.01 (0.11) | 0.01 (0.07) | 0.01 (0.07) | 0.00 (0.07) | 0.01 (0.07) |
| Residual | 1.17 (1.08) | 1.16 (1.08) | 1.17 (1.08) | 1.17 (1.08) | 1.93 (1.39) | 1.93 (1.39) | 1.93 (1.39) | 1.93 (1.39) |

***Note.*** Est. = Estimate; SE = Standard Error; Day’s Connect = Daily P-A connectedness; Day’s Conflict = Daily P-A conflict; GConnect = General P-A connectedness; GConflict = General P-A conflict; ** p < .01, * p < .05

**Table S3.** Full Model for General Parent-Adolescent Conflict

|  | *Negative Mood* | *Positive Mood* |
| --- | --- | --- |
| Fixed Effects | *Est. (SE)* | *Est. (SE)* |
| Intercept | 1.12 (0.18) ** | 8.35 (0.21) ** |
| Day’s Connect | -0.15 (0.03) ** | 0.30 (0.04) ** |
| Day’s Conflict | 0.11 (0.02) ** | -0.08 (0.02) ** |
| Day of Study | -0.02 (0.00) ** | 0.01 (0.01) |
| Adol Sex | 0.45 (0.23) * | -0.47 (0.26) |
| G­­Connect | -0.52 (0.18) ** | 1.06 (0.20) ** |
| GConflict | 0.82 (0.17) ** | -0.28 (0.20) |
| Day’s Conflict * GConflict | 0.05 (0.03) * | 0.00 (0.03) |
| Day’s Connect* GConflict | -0.00 (0.04) | 0.02 (0.05) |
| Random Effects |  |  |
| Intercept | 1.75 (1.32) | 2.33 (1.53) |
| Day’s Connect | 0.05 (0.22) | 0.06 (0.25) |
| Day’s Conflict | 0.01 (0.10) | 0.00 (0.07) |
| Residual | 1.23 (1.11) | 2.00 (1.41) |

***Note.*** Est. = Estimate; SE = Standard Error; Day’s Connect = Daily P-A connectedness; Day’s Conflict = Daily P-A conflict; GConnect = General P-A connectedness; GConflict = General P-A conflict; ** p < .01, * p < .05

**Table S4.** Full Model for General Parent-Adolescent Connectedness

|  | *Negative Mood* | *Positive Mood* |
| --- | --- | --- |
| Fixed Effects | *Est. (SE)* | *Est. (SE)* |
| Intercept | 1.12 (0.18) ** | 8.35 (0.21) ** |
| Day’s Connect | -0.15 (0.03) ** | 0.30 (0.04) ** |
| Day’s Conflict | 0.12 (0.02) ** | -0.09 (0.02) ** |
| Day of Study | -0.02 (0.00) ** | 0.01 (0.01) |
| Adol Sex | 0.45 (0.23) * | -0.48 (0.26) |
| G­­Connect | -0.54 (0.18) ** | 1.02 (0.20) ** |
| GConflict | 0.80 (0.17) ** | -0.29 (0.20) |
| Day’s Conflict * GConnect | -0.06 (0.03) * | -0.04 (0.03) |
| Day’s Connect * GConnect | 0.03 (0.05) | -0.01 (0.06) |
| Random Effects |  |  |
| Intercept | 1.75 (1.32) | 2.34 (1.53) |
| Day’s Connect | 0.05 (0.22) | 0.06 (0.25) |
| Day’s Conflict | 0.01 (0.10) | 0.00 (0.06) |
| Residual | 1.23 (1.11) | 2.00 (1.41) |

***Note.*** Est. = Estimate; SE = Standard Error; Day’s Connect = Daily P-A connectedness; Day’s Conflict = Daily P-A conflict; GConnect = General P-A connectedness; GConflict = General P-A conflict; ** p < .01, * p < .05

**Table S5.** Results of False Discovery Rate (FDR) Correction for Cross-Level Interactions

| **Outcome** | **Interaction Term** | **Est.** | **SE** | **t** | **Original *p*** | **FDR-adjusted *p*** |
| --- | --- | --- | --- | --- | --- | --- |
| Negative Affect | Day’s Conflict × General Conflict | 0.05 | 0.03 | 2.10 | 0.036 | 0.055 |
| Negative Affect | Day’s Connect × General Conflict | -0.02 | 0.04 | -0.58 | 0.562 | 0.590 |
| Negative Affect | Day’s Conflict × General Connect | -0.07 | 0.03 | -2.58 | 0.010 | 0.016 |
| Negative Affect | Day’s Connect × General Connect | 0.06 | 0.05 | 1.20 | 0.229 | 0.244 |
| Positive Affect | Day’s Conflict × General Conflict | 0.00 | 0.03 | 0.09 | 0.927 | 0.927 |
| Positive Affect | Day’s Connect × General Conflict | 0.02 | 0.05 | 0.38 | 0.707 | 0.730 |
| Positive Affect | Day’s Conflict × General Connect | -0.04 | 0.03 | -1.29 | 0.196 | 0.213 |
| Positive Affect | Day’s Connect × General Connect | 0.01 | 0.06 | 0.22 | 0.828 | 0.841 |

***Note.*** Est. = Estimate; SE = Standard Error; Day’s Connect = Daily P-A connectedness; Day’s Conflict = Daily P-A conflict; General Connect = General P-A connectedness; General Conflict = General P-A conflict.

**Table S6.** Models Adding Prior-Day Outcome

|  | *Negative Mood* | | | | *Positive Mood* | | | |
| --- | --- | --- | --- | --- | --- | --- | --- | --- |
| Fixed Effects | *Est. (SE)* | *Est. (SE)* | *Est. (SE)* | *Est. (SE)* | *Est. (SE)* | *Est. (SE)* | *Est. (SE)* | *Est. (SE)* |
| Intercept | 0.80 (0.13) ** | 0.80 (0.13) ** | 0.80 (0.13) ** | 0.80 (0.13) ** | 6.69 (0.23) ** | 6.66 (0.23) ** | 6.69 (0.23) ** | 6.69 (0.23) ** |
| Day’s Connect | -0.16 (0.03) ** | -0.16 (0.04) ** | -0.16 (0.03) ** | -0.15 (0.04) ** | 0.30 (0.04) ** | 0.30 (0.04) ** | 0.30 (0.04) ** | 0.30 (0.04) ** |
| Day’s Conflict | 0.12 (0.02) ** | 0.13 (0.02) ** | 0.12 (0.02) ** | 0.13 (0.02) ** | -0.08 (0.03) ** | -0.08 (0.02) ** | -0.08 (0.02) ** | -0.08 (0.02) ** |
| Day of Study | -0.01 (0.00) ** | -0.01 (0.00) ** | -0.01 (0.00) ** | -0.01 (0.00) ** | 0.01 (0.00) | 0.01 (0.00) | 0.01 (0.00) | 0.01 (0.00) |
| Adol Sex | 0.30 (0.16) | 0.30 (0.16) | 0.30 (0.17) | 0.30 (0.16) | -0.38 (0.21) | -0.38 (0.21) | -0.39 (0.21) | -0.38 (0.21) |
| Prior-Day Outcome | 0.27 (0.02) ** | 0.27 (0.02) ** | 0.27 (0.02) ** | 0.27 (0.02) ** | 0.20 (0.02) ** | 0.20 (0.02) ** | 0.20 (0.02) ** | 0.20 (0.02) ** |
| GConnect | -0.38 (0.13) ** | -0.38 (0.13) ** | -0.39 (0.13) ** | -0.38 (0.13) ** | 0.85 (0.17) ** | 0.85 (0.16) ** | 0.83 (0.17) ** | 0.85 (0.17) ** |
| GConflict | 0.61 (0.13) ** | 0.60 (0.13) ** | 0.60 (0.13) ** | 0.60 (0.13) ** | -0.24 (0.16) | -0.25 (0.16) | -0.26 (0.16) | -0.25 (0.16) |
| Day’s Conflict * GConflict | 0.05 (0.03) † | -- | -- | -- | 0.01 (0.03) | -- | -- | -- |
| Day’s Connect * GConflict | -- | 0.00 (0.04) | -- | -- | -- | 0.02 (0.05) | -- | -- |
| Day’s Conflict * GConnect | -- | -- | -0.06 (0.03) * | -- | -- | -- | -0.04 (0.03) | -- |
| Day’s Connect * GConnect | -- | -- | -- | 0.03 (0.05) | -- | -- | -- | -0.00 (0.06) |
| Random Effects |  |  |  |  |  |  |  |  |
| Intercept | 0.90 (0.95) | 0.91 (0.95) | 0.92 (0.96) | 0.91 (0.95) | 1.53 (1.24) | 1.51 (1.23) | 1.53 (1.23) | 1.52 (1.23) |
| Day’s Connect | 0.05 (0.23) | 0.05 (0.23) | 0.05 (0.23) | 0.05 (0.23) | 0.06 (0.25) | 0.06 (0.25) | 0.06 (0.25) | 0.06 (0.25) |
| Day’s Conflict | 0.01 (0.10) | 0.01 (0.11) | 0.01 (0.10) | 0.01 (0.11) | 0.01 (0.08) | 0.01 (0.07) | 0.00 (0.07) | 0.01 (0.07) |
| Residual | 1.15 (1.07) | 1.15 (1.07) | 1.15 (1.07) | 1.15 (1.07) | 1.93 (1.39) | 1.93 (1.39) | 1.93 (1.39) | 1.93 (1.39) |

***Note.*** Est. = Estimate; SE = Standard Error; Day’s Connect = Daily P-A connectedness; Day’s Conflict = Daily P-A conflict; Adol Sex = Adolescent sex; Prior-Day Outcome = the previous day’s negative (or positive) mood; GConnect = General P-A connectedness; GConflict = General P-A conflict. ** p < .01, * p < .05. † p = .076

**Table S7.** Correlations Between Family-Level and Parent-Adolescent Relationships

| Variable | Parent-Adolescent Connectedness | Parent-Adolescent Conflict | Family Cohesion | Family Conflict |
| --- | --- | --- | --- | --- |
| 1. Parent-Adolescent Connectedness | -- | -.58** | 0.81** | -0.42** |
| 2. Parent-Adolescent Conflict | -.43** | -- | -0.49** | 0.73** |
| 3. Family Cohesion | .52** | -.25** | -- | -.64** |
| 4. Family Conflict | -.29** | .39** | -.38** | -- |

***Note.*** ** p < .01, * p < .05. Between-person (average) correlations are shown above the diagonal; within-person (daily) correlations are shown below the diagonal.

**Family cohesion.** In the daily survey, adolescents reported on family cohesion using three items selected from the short version of the Family Environment Scale (Moos & Moos, 1994): “Family members really helped and supported one another,” “There was a feeling of togetherness in our family,” and “Family members really backed each other up.” Responses to the three items were averaged to create a daily family cohesion scale, with higher scores reflecting greater family cohesion. Adolescents rated each item on a 10-point scale, with the option to adjust in 0.1 increments (*M* = 7.87, *SD* = 2.41). In this sample, this scale exhibited good within-person and between-person reliability ($R_{1F}$ = 0.92, $R_{c}$ = 0.77).

**Family conflict**. Daily family conflict was assessed using two items drawn from the shortened Family Environment Scale (Moos & Moos, 1994): “Family members criticized one another" and "Family members fought.” These two items were averaged to form a daily family conflict scale, with higher scores reflecting greater family conflict. Adolescents rated each item on a 10-point scale that ranged, with the option to adjust in 0.1 increments (*M* = 1.45, *SD* = 2.26). In this sample, this scale exhibited good within-person and between-person reliability ($R_{1F}$ = 0.78, $R_{c}$ = 0.70).

**Table S8**. Models Testing Additional Covariates

|  | *Negative Mood* | | | | *Positive Mood* | | | |
| --- | --- | --- | --- | --- | --- | --- | --- | --- |
| Fixed Effects | *Est. (SE)* | *Est. (SE)* | *Est. (SE)* | *Est. (SE)* | *Est. (SE)* | *Est. (SE)* | *Est. (SE)* | *Est. (SE)* |
| Intercept | -0.61 (1.48) | -0.57 (1.48) | -0.60 (1.48) | -0.58 (1.48) | 6.91 (1.70) ** | 6.92 (1.70) ** | 6.91 (1.70) ** | 6.91 (1.70) ** |
| Day’s PA Connect | -0.08 (0.03) * | -0.07 (0.03) * | -0.08 (0.03) * | -0.07 (0.03) * | 0.19 (0.04) ** | 0.19 (0.04) ** | 0.19 (0.04) ** | 0.20 (0.04) ** |
| Day’s PA Conflict | 0.07 (0.02) ** | 0.09 (0.02) ** | 0.08 (0.02) ** | 0.09 (0.02) ** | -0.06 (0.02) * | -0.06 (0.02) ** | -0.07 (0.02) ** | -0.06 (0.02) ** |
| Day’s Fam Coh | -0.11 (0.02) ** | -0.11 (0.02) ** | -0.11 (0.02) ** | -0.11 (0.02) ** | 0.20 (0.03) ** | 0.20 (0.03) ** | 0.20 (0.03) ** | 0.20 (0.03) ** |
| Day’s Fam Conflict | 0.09 (0.02) ** | 0.10 (0.02) ** | 0.09 (0.02) ** | 0.09 (0.02) ** | -0.04 (0.02) * | -0.04 (0.02) * | -0.04 (0.02) * | -0.04 (0.02) * |
| Day of Study | -0.02 (0.00) ** | -0.02 (0.00) ** | -0.02 (0.00) ** | -0.02 (0.00) ** | 0.01 (0.01) | 0.01 (0.01) | 0.01 (0.01) | 0.01 (0.01) |
| School Day | 0.18 (0.04) ** | 0.18 (0.04) ** | 0.18 (0.04) ** | 0.18 (0.04) ** | -0.03 (0.06) | -0.03 (0.06) | -0.03 (0.06) | -0.03 (0.06) |
| Completed Days | -0.08 (0.03) * | -0.08 (0.03) ** | -0.08 (0.03) * | -0.08 (0.03) * | 0.13 (0.04) ** | 0.13 (0.04) ** | 0.13 (0.04) ** | 0.13 (0.04) ** |
| Adol Age | 0.10 (0.10) | 0.10 (0.10) | 0.10 (0.10) | 0.10 (0.10) | -0.03 (0.11) | -0.03 (0.11) | -0.03 (0.11) | -0.03 (0.11) |
| Adol Sex | 0.33 (0.16) * | 0.33 (0.16) * | 0.33 (0.16) * | 0.33 (0.16) * | -0.24 (0.18) | -0.24 (0.18) | -0.24 (0.18) | -0.24 (0.18) |
| Adol Dep | 1.29 (0.15) ** | 1.28 (0.15) ** | 1.28 (0.15) ** | 1.28 (0.15) ** | -0.99 (0.17) ** | -0.99 (0.17) ** | -0.99 (0.17) ** | -1.00 (0.17) ** |
| Parent Sex | -0.13 (0.36) | -0.13 (0.37) | -0.13 (0.36) | -0.13 (0.37) | 0.81 (0.42) | 0.81 (0.42) | 0.81 (0.42) | 0.82 (0.42) |
| G Fam Coh | 0.09 (0.05) | 0.09 (0.05) | 0.09 (0.05) | 0.09 (0.05) | 0.47 (0.06) ** | 0.47 (0.06) ** | 0.47 (0.06) ** | 0.47 (0.06) ** |
| G Fam Conflict | 0.46 (0.06) ** | 0.46 (0.06) ** | 0.45 (0.06) ** | 0.45 (0.06) ** | -0.03 (0.07) | -0.03 (0.07) | -0.03 (0.07) | -0.03 (0.07) |
| G PA Connect | -0.33 (0.13) * | -0.34 (0.13) * | -0.36 (0.13) ** | -0.34 (0.13) * | 0.27 (0.15) | 0.26 (0.15) | 0.25 (0.15) | 0.26 (0.15) |
| G PA Conflict | 0.03 (0.14) | 0.00 (0.14) | -0.00 (0.14) | 0.00 (0.14) | 0.24 (0.16) | 0.24 (0.16) | 0.24 (0.16) | 0.24 (0.16) |
| Day’s PA Conflict * GConflict | 0.05 (0.02) * | -- | -- | -- | 0.00 (0.03) | -- | -- | -- |
| Day’s PA Connect * G PA Conflict | -- | -0.03 (0.04) | -- | -- | -- | 0.02 (0.05) | -- | -- |
| Day’s PA Conflict * G PA Connect | -- | -- | -0.05 (0.03)* | -- | -- | -- | -0.04 (0.03) | -- |
| Day’s PA Connect * G PA Connect | -- | -- | -- | 0.04 (0.05) | -- | -- | -- | 0.04 (0.05) |
| Random Effects |  |  |  |  |  |  |  |  |
| Intercept | 0.73 (0.85) | 0.73 (0.85) | 0.73 (0.85) | 0.73 (0.85) | 0.95 (0.97) | 0.95 (0.97) | 0.95 (0.98) | 0.95 (0.97) |
| Day’s Connect | 0.05 (0.22) | 0.05 (0.22) | 0.05 (0.22) | 0.05 (0.22) | 0.06 (0.24) | 0.06 (0.24) | 0.06 (0.24) | 0.06 (0.24) |
| Day’s Conflict | 0.01 (0.09) | 0.01 (0.10) | 0.01 (0.09) | 0.01 (0.10) | 0.00 (0.04) | 0.00 (0.04) | 0.00 (0.04) | 0.00 (0.04) |
| Residual | 1.15 (1.07) | 1.15 (1.07) | 1.15 (1.07) | 1.15 (1.07) | 1.89 (1.37) | 1.89 (1.37) | 1.88 (1.37) | 1.88 (1.37) |

***Note.*** Est. = Estimate; SE = Standard Error; Day’s PA Connect = Daily P-A connectedness; Day’s PA Conflict = Daily P-A conflict; Day’s Fam Coh = Daily family cohesion; Day’s Fam Conflict = Daily family conflict; Adol = Adolescent; Adol Dep = Adolescent baseline depressive symptoms. **G Fam** Coh = General family cohesion; G Fam Conflict = General family conflict; G PA Connect = General P-A connectedness; G PA Conflict = General P-A conflict; ** p < .01, * p < .05.

**Adolescent baseline depressive symptoms.** Adolescent baseline depressive symptoms were assessed using the 10-item Depression Scale from the Revised Child Anxiety and Depression Scale (Chorpita et al., 2000). An example item is, “Nothing is much fun anymore.” Items were rated on a 4-point scale from 1 (Never) to 4 (Always) and were averaged to create a total depressive symptoms score, with higher scores indicating higher levels of depressive symptoms (*M* = 1.52, *SD* = 0.57). The scale has demonstrated strong reliability and validity in prior research (Chorpita et al., 2000), and showed excellent internal reliability in the present sample (Cronbach’s α = .90).

**References**

Chorpita, B. F., Yim, L., Moffitt, C., Umemoto, L. A., & Francis, S. E. (2000). Assessment of symptoms of DSM-IV anxiety and depression in children: a revised child anxiety and depression scale. Behaviour Research and Therapy, 38(8), 835–855. https:// doi.org/10.1016/S0005-7967(99)00130-8.

Moos, R. H., & Moos, B. S. (1994). *Family environment scale manual.* Consulting Psychologists Press.
